# Effects of risk factors on the development and mortality of early- and late-onset dementia: an 11-year longitudinal nationwide population-based cohort study in South Korea

**DOI:** 10.1186/s13195-024-01436-5

**Published:** 2024-04-25

**Authors:** Min Young Chun, Wonjeong Chae, Sang Won Seo, Hyemin Jang, Jihwan Yun, Duk L. Na, Dongwoo Kang, Jungkuk Lee, Dustin B. Hammers, Liana G. Apostolova, Sung-In Jang, Hee Jin Kim

**Affiliations:** 1 Department of Neurology, Samsung Medical Center, Sungkyunkwan University School of Medicine, 81 Irwon-ro, Gangnam-gu, Seoul, 06351 South Korea; 2 Department of Neurology, Yonsei University College of Medicine, 50-1, Yonsei-Ro, Seodaemun-gu, Seoul, 03722 South Korea; 3 Department of Neurology, Yongin Severance Hospital, Yonsei University Health System, 363 Dongbaekjukjeon-daero, Giheung-gu, , Yongin-si, Gyeonggi-do 16995 South Korea; 4 Office of Strategic Planning, Healthcare Policy and Strategy Task Force, Yonsei University Health System, 50-1, Yonsei-Ro, Seodaemun-gu, Seoul, 03722 South Korea; 5 Alzheimer’s Disease Convergence Research Center, Samsung Medical Center, 81 Irwon-ro, Gangnam-gu, Seoul, 06351 South Korea; 6 Department of Health Sciences and Technology, SAIHST, Sungkyunkwan University, 81 Irwon-ro, Gangnam-gu, Seoul, 06351 South Korea; 7 Department of Digital Health, SAIHST, Sungkyunkwan University, 81 Irwon-ro, Gangnam-gu, Seoul, 06351 South Korea; 8 Department of Neurology, Seoul National University Hospital, 101 Daehak-ro, Jongno-gu, Seoul, South Korea; 9 Department of Neurology, Soonchunhyang University Bucheon Hospital, 170, Jomaru-ro, Wonmi-Gu, Bucheon-si, Gyeonggi-do, 14574 South Korea; 10 Department of Data Science, Hanmi Pharm. Co., Ltd, 14, Wiryeseong-daero, Songpa-gu, Seoul, South Korea; 11 Department of Neurology, Indiana University School of Medicine, 355 W 16th St, Indianapolis, IN USA; 12 Department of Radiology and Imaging Sciences, Center for Neuroimaging, Indiana University School of Medicine Indianapolis, 355W 16th St, Indianapolis, IN USA; 13 Department of Medical and Molecular Genetics, Indiana University School of Medicine, 355W 16th St, Indianapolis, IN USA; 14 Department of Preventive Medicine, College of Medicine, Yonsei University, 50-1, Yonsei-Ro, Seodaemun-gu, Seoul, 03722 South Korea

**Keywords:** Dementia, Onset age, Development, Mortality, Risk factor, Population study

## Abstract

**Background:**

Early-onset dementia (EOD, onset age < 65) and late-onset dementia (LOD, onset age ≥ 65) exhibit distinct features. Understanding the risk factors for dementia development and mortality in EOD and LOD respectively is crucial for personalized care. While risk factors are known for LOD development and mortality, their impact on EOD remains unclear. We aimed to investigate how hypertension, diabetes mellitus, hyperlipidemia, atrial fibrillation, and osteoporosis influence the development and mortality of EOD and LOD, respectively.

**Methods:**

Using the Korean National Health Insurance Service (NHIS) database, we collected 546,709 dementia-free individuals and followed up for 11 years. In the two study groups, the Younger group (< 65 years old) and the Older group (≥ 65 years old), we applied Cox proportional hazard models to assess risk factors for development of EOD and LOD, respectively. Then, we assessed risk factors for mortality among EOD and LOD.

**Results:**

Diabetes mellitus and osteoporosis increased the risk of EOD and LOD development. Hypertension increased the risk of EOD, while atrial fibrillation increased the risk of LOD. Conversely, hyperlipidemia exhibited a protective effect against LOD development. Additionally, diabetes mellitus increased mortality in EOD and LOD. Hypertension and atrial fibrillation increased mortality in LOD, while hyperlipidemia decreased mortality in EOD and LOD.

**Conclusions:**

Risk factors influencing dementia development and mortality differed in EOD and LOD. Targeted public health interventions addressing age-related risk factors may reduce dementia incidence and mortality.

**Supplementary Information:**

The online version contains supplementary material available at 10.1186/s13195-024-01436-5.

## Background

Dementia has emerged as a worldwide public health concern and socioeconomic burden due to its extensive impacts on patients, families, and society [1]. Understanding the risk factors contributing to dementia development and mortality is crucial to prevent and manage the condition, which is currently the seventh leading cause of death worldwide [2, 3]. Previous studies have demonstrated that several modifiable risk factors for developing dementia include hypertension, diabetes mellitus, hyperlipidemia, atrial fibrillation, and osteoporosis [4–10]. These risk factors also have a detrimental effect on the progression of dementia and lead to an increased rate of decline and mortality.

Dementia has different clinical characteristics according to the onset of age. Approximately 5% of dementia patients develop their symptoms before age 65 (early-onset dementia, EOD) [10, 11]. While late-onset dementia (LOD, dementia onset ≥ 65 years old) patients show memory impairment and slow decline, EOD patients show more non-memory symptoms (e.g., executive dysfunction, visuospatial dysfunction, and behavioral symptoms) and rapid decline [12–14]. Underlying etiologies and neuropathological findings of EOD and LOD tend to be different [15–19]. Risk factors for developing LOD include hypertension, diabetes, atrial fibrillation, *APOE* ε4 allele, and low education [20, 21]. However, only a few studies have investigated the risk factors for developing EOD [22]. Furthermore, risk factors for mortality among EOD and LOD are not well known.

A nationwide population-based cohort study offers distinct advantages over smaller-scale studies, including a larger sample size and its ability to reflect the characteristics of the entire nation more accurately. While prior studies in several countries have explored the risk factors associated with dementia using nationwide population-based cohorts [23–25], this is the first study to investigate how these risk factors impact dementia development and mortality according to age. Such identification of age-related dementia risk factors can contribute to the development of targeted nationwide health programs based on patient age, which have the potential to reduce treatment costs and lessen the burden on caregivers.

Therefore, we aimed to investigate the effect of a selection of known LOD risk factors (hypertension, diabetes mellitus, hyperlipidemia, atrial fibrillation, and osteoporosis) on the incidence and mortality of both EOD and LOD by utilizing the nationwide population-based cohort study of South Korea. Given the distinct clinical profiles of EOD and LOD, we hypothesized that the influence of these risk factors varies with the age of dementia onset.

## Methods

### Data source and study population

We used the Korean National Health Insurance Service (NHIS) database, which contains primary demographic and health insurance claim data from 2002. The NHIS is a single insurer under the universal health insurance service in which all Korean citizens are enrolled. The NHIS database contains annual data on diagnoses and medications that allow us to track the incidence of dementia over time. De-identified NHIS data is available on request from a government agency for research purposes via the website (http://nhiss.nhis.or.kr).

This study was approved by the NHIS (NHIS-2021-1-231) and the Institutional Review Board of Samsung Medical Center, Seoul, Korea (SMC IRB 2022-03-029). Informed consent was waived due to the retrospective nature of the study and the restricted use of anonymous data.

**Fig. 1:**
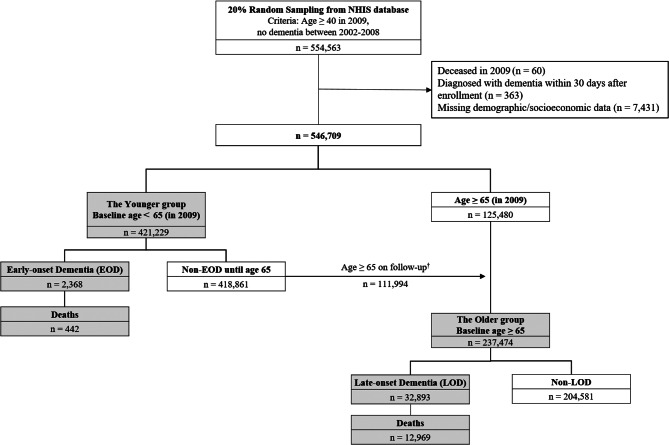
Flow chart of the study population. ^†^Among the 418,861 individuals who did not develop EOD until age 65, 114,288 individuals reached 65 years of age during the study window. After excluding individuals with missing data for demographic/socioeconomic (*n* = 2,188) and deceased/censored/diagnosed with dementia within 30 days after entry to the older group (*n* = 106), 111,994 individuals were transitioned from the Younger group to the Older group

We selected 2009 as the baseline year to begin our 11-year study window, and we considered annual data from 2002 to 2008 as the run-in window for determining dementia-free status. From the 2009 NHIS database, we extracted 20% of the Korean population through a systematic stratified random sampling method. This extraction value was determined based on processing limitations due to the volume of information present in each yearly entry. From the 20% extraction, we established a dementia-free cohort (*n* = 554,563) with the following criteria: (1) individuals who were 40 years or older in 2009, and (2) individuals who had not been diagnosed with dementia or prescribed with dementia medication between 2002 and 2008. This 7-year run-in period was applied to ensure that individuals were stable and without dementia at the baseline of the observation period (Fig. 1). Then, we excluded 60 individuals who died in 2009 and 363 individuals who were diagnosed with dementia within 30 days of enrollment in 2009. In addition, we excluded 7,431 individuals without demographic or socioeconomic data. A total of 546,709 individuals were included in the Korean dementia-free cohort at baseline in 2009 (Fig. 1).

We created two dementia-free groups: the Younger group (< 65 years old) and the Older group (≥ 65 years old) (Fig. 1). The Younger group consisted of 421,299 individuals aged under 65 years in 2009. Individuals were monitored until they reached the age of 65 and those who developed dementia were classified as having EOD. At the start of the study in 2009, 125,480 individuals belonged to the Older group. Individuals who originally belonged to the Younger group at the start of the study and remained dementia-free during the study period until they reached the age of 65 (*n* = 111,994) were then transferred from the Younger group to the Older group. In these cases, their data at age 65 were used as baseline demographics for the Older group analyses. The Older group consisted of 237,474 individuals, from which we identified those who developed LOD.

### Outcome variables

Participants were observed for dementia incidence and mortality over 11 years from 2009 to 2019.

#### Definition of dementia

To identify individuals with dementia, we utilized a combination of diagnostic codes -International Statistical Classification of Diseases and Related Health Problems, 10th revision (ICD-10)- and prescription records. The age at which both diagnostic codes for Alzheimer’s dementia (e.g., F00.0, F00.1, F00.2, F00.9, G30.0, G30.1, G30.8, and G30.9) and prescriptions for Alzheimer’s dementia medication (e.g., donepezil, galantamine, rivastigmine, memantine) were initially recorded would be considered as their onset age (Supplementary Table 1). EOD was defined as age-at-diagnosis < 65, and LOD as age-at-diagnosis ≥ 65.

#### Mortality

The date of death was taken from the Korean NHIS database. We determined the number of deaths in each group of EOD and LOD. Time to death was calculated as the years from age at onset of dementia to death. We examined all-cause mortality in relation to risk factors in EOD and LOD.

### Independent variables

The study included hypertension, diabetes mellitus, atrial fibrillation, hyperlipidemia, and osteoporosis as risk factors of interest. For other possible confounders, we included other risk factors of Charlson’s comorbidity: myocardial infarction, congestive heart failure, peripheral vascular disease, cerebrovascular disease, chronic pulmonary disease, rheumatic or connective tissue disease, gastric or peptic ulcer, liver disease, hemiplegia or paraplegia, and moderate or severe renal disease. The presence of the above factors was defined using the ICD-10 codes. Demographics such as age, sex, residential area, and socioeconomic status were also adjusted in the study. The residential areas were divided into two groups: urban areas (Seoul, Busan, Daegu, Daejeon, Gwangju, Incheon, and Ulsan) and rural areas (Gyeonggi, Gangwon, Chungcheongbuk, Chungcheongnam, Jeollabuk, Jeollanam, Gyeongsangbuk, Gyeongsangnam, and Jeju). The socioeconomic status was divided into the top 20% of the population and the rest based on the amount of health insurance tax.

### Statistical analyses

Chi-square tests and univariate analysis were used for descriptive statistics in each age group (Tables 1 and 2). For considering the primary analyses, we started with a cohort of dementia-free participants and tracked their NHIS data annually to identify those who developed dementia. To investigate the effect of each risk factor on dementia development and all-cause mortality, 1:N propensity score matching (PSM) based on age and sex was applied from the dementia-free cohort. The PSM was conducted for each risk factor, considering the number of individuals with each risk factor of interest varied for each specific risk factor.

**Table 1:** Clinical characteristics of Younger group and Older group

Variables	**Younger group (Age < 65)**						Older group (Age ≥ 65)					
	Total Number	EOD (n)	%	Non-EOD (n)	%	*p* value	Total Number	LOD (n)	%	Non-LOD (n)	%	*p* value
**Total Participants**	421,229	2,368	0.6	418,861	99.4		237,474	32,893	13.9	204,581	86.1	
**Age**						< 0.001						< 0.001
Mean ± Standard deviation		53.0 ± 5.2		50.3 ± 6.8				74.2 ± 6.6		68.3 ± 5.4		
**Sex**						0.002						< 0.001
Male	206,289	1,236	52.2	205,053	49.0		105,868	10,725	32.6	95,143	46.5	
Female	214,940	1,132	47.8	213,808	51.0		131,606	22,168	67.4	109,438	53.5	
**Risk factors of interest**												
Hypertension	115,954	1,057	44.6	114,897	27.4	< 0.001	154,574	23,901	72.7	130,673	63.9	< 0.001
Diabetes mellitus	89,105	910	38.4	88,195	21.1	< 0.001	117,142	17,143	52.1	99,999	48.9	< 0.001
Atrial fibrillation	5,269	54	2.3	5,215	1.2	< 0.001	9,940	1,564	4.8	8,376	4.1	< 0.001
Hyperlipidemia	123,711	989	41.8	122,722	29.3	< 0.001	138,984	16,756	50.9	122,228	59.7	< 0.001
Osteoporosis	49,302	417	17.6	48,885	11.7	< 0.001	90,494	15,689	47.7	74,805	36.6	< 0.001
**Other risk factors in the Charlson comorbidity index**												
Myocardial infarct	18,267	198	8.4	18,069	4.3	< 0.001	33,889	5,319	16.2	28,570	14.0	< 0.001
Congestive heart failure	7,121	93	3.9	7,028	1.7	< 0.001	19,661	3,683	11.2	15,978	7.8	< 0.001
Peripheral vascular disease	55,432	557	23.5	54,875	13.1	< 0.001	91,160	13,832	42.1	77,328	37.8	< 0.001
Cerebrovascular disease	24,434	499	21.1	23,935	5.7	< 0.001	52,585	9,685	29.4	42,900	21.0	< 0.001
Chronic pulmonary disease	146,184	933	39.4	145,251	34.7	< 0.001	138,475	19,467	59.2	119,008	58.2	< 0.001
Rheumatic or connective tissue disease	6,623	40	1.7	6,583	1.6	0.647	7,123	925	2.8	6,198	3.0	0.032
Gastric or peptic ulcer	157,368	1,000	42.2	156,368	37.3	< 0.001	135,912	17,723	53.9	118,189	57.8	< 0.001
Mild liver disease	180,848	1,354	57.2	179,494	42.9	< 0.001	145,014	18,247	55.5	126,767	62.0	< 0.001
Hemiplegia or paraplegia	3,221	152	6.4	3,069	0.7	< 0.001	6,223	1,333	4.1	4,890	2.4	< 0.001
Moderate or severe renal disease	5,535	72	3.0	5,463	1.3	< 0.001	9,301	1,236	3.8	8,065	3.9	0.109
**Socioeconomic status**						< 0.001						< 0.001
Upper 20%	121,591	400	16.9	121,191	28.9		72,803	10,797	32.8	62,006	30.3	
Lower 80%	299,638	1,968	83.1	297,670	71.1		164,671	22,096	67.2	142,575	69.7	
**Residential area**						< 0.001						< 0.001
Urban	199,689	1,011	42.7	198,678	47.4		102,405	12,191	37.1	90,214	44.1	
Rural	221,540	1,357	57.3	220,183	52.6		135,069	20,702	62.9	114,367	55.9	

*Abbreviations*: EOD, early-onset dementia; LOD, late-onset dementia

**Table 2:** All-cause mortality and related factors among early-onset dementia and late-onset dementia individuals

Variables	Early-onset dementia (n = 2,368)					Late-onset dementia (n = 32,893)				
	Death (n)	%	Non-death (n)	%	*p* value	Death (n)	%	Non-death (n)	%	*p* value
**Total Participants**	442	18.7	1,926	81.3		12,969	39.4	19,924	60.6	
**Age**					< 0.001					< 0.001
Mean ± Standard deviation	54.8 ± 5.2		52.6 ± 5.1			77.2 ± 6.8		72.1 ± 5.76		
**Sex**					< 0.001					< 0.001
Male	310	70.1	926	48.1		5,087	39.2	5,638	28.3	
Female	132	29.9	1,000	51.9		7,882	60.8	14,286	71.7	
**Risk factors of interest**										
Hypertension	224	50.7	833	43.3	0.005	9,782	75.4	14,119	70.9	< 0.001
Diabetes mellitus	205	46.4	705	36.6	< 0.001	6,832	52.7	10,311	51.8	0.100
Atrial fibrillation	16	3.6	38	2.0	0.037	752	5.8	812	4.1	< 0.001
Hyperlipidemia	179	40.5	810	42.1	0.549	5,991	46.2	10,765	54.0	< 0.001
Osteoporosis	54	12.2	363	18.8	0.001	5,806	44.8	9,883	49.6	< 0.001
**Other risk factors in the Charlson comorbidity index**										
Myocardial infarct	59	13.3	139	7.2	< 0.001	2,293	17.7	3,026	15.2	< 0.001
Congestive heart failure	31	7.0	62	3.2	< 0.001	1,785	13.8	1,898	9.5	< 0.001
Peripheral vascular disease	105	23.8	452	23.5	0.898	5,407	41.7	8,425	42.3	0.286
Cerebrovascular disease	110	24.9	389	20.2	0.029	4,083	31.5	5,602	28.1	< 0.001
Chronic pulmonary disease	166	37.6	767	39.8	0.379	7,716	59.5	11,751	59.0	0.352
Rheumatic or connective tissue disease	10	2.3	30	1.6	0.300	346	2.7	579	2.9	0.202
Gastric or peptic ulcer	178	40.3	822	42.7	0.355	6,494	50.1	11,229	56.4	< 0.001
Mild liver disease	254	57.5	1,100	57.1	0.892	6,713	51.8	11,534	57.9	< 0.001
Hemiplegia or paraplegia	45	10.2	107	5.6	< 0.001	634	4.9	699	3.5	< 0.001
Moderate or severe renal disease	28	6.3	44	2.3	< 0.001	628	4.8	608	3.1	< 0.001
**Socioeconomic status**					0.348					0.848
Upper 20%	68	15.4	332	17.2		4,265	32.9	6,532	32.8	
Lower 80%	374	84.6	1,594	82.8		8,704	67.1	13,392	67.2	
**Residential area**					0.975					0.618
Urban	189	42.8	822	42.7		4,828	37.2	7,363	37.0	
Rural	253	57.2	1,104	57.3		8,141	62.8	12,561	63.0	

To estimate the hazard ratios (HRs) of each risk factor of interest in the development of EOD and LOD, we applied the multivariate Cox proportional hazard model, which included age, sex, risk factor of interest (hypertension, diabetes, atrial fibrillation, hyperlipidemia, and osteoporosis), other risk factors in Charlson comorbidity index, socioeconomic status, and residential area as independent variables.

To understand the development of mortality among EOD and LOD patients, a comparable set of analyses was used as described immediately above – with mortality being the outcome variable. Survival curves were estimated by the Kaplan–Meier method. To estimate the HRs of each risk factor of interest for mortality in EOD and LOD patients, we used multivariate Cox proportional hazard models including the same independent variables as described above.

All analyses were performed using SAS software (version 9.4; SAS Institute, Cary, North Carolina, USA).

## Results

### Study population

Clinical characteristics of Younger group and Older group are shown in Table 1. Overall, among 546,709 individuals, 35,261 (6.4%) developed dementia (Table 1), and among those with dementia, 13,411 (38.0%) died during the observation period of 11 years from 2009 to 2019 (Table 2). In the Younger group (< 65 years old) of 421,229 individuals, 2,368 (0.6%) developed dementia (i.e., EOD). The number of deaths among the EOD group was 442 (18.7%). Conversely, in the Older group (≥ 65 years old) of 237,474 individuals, 32,893 subjects (13.9%) developed LOD, in which 12,969 patients (39.4%) died within the observation period of 11 years.

In the Younger group, those who developed EOD were older at baseline (EOD 53.0 ± 5.2 years vs. non-EOD 50.3 ± 6.8 years, *p* < 0.001) and more likely to be male (EOD 52.2% vs. non-EOD 49.0%, *p* = 0.002) than those who did not develop dementia. The most prevalent risk factor in EOD was hypertension (44.6%), followed by hyperlipidemia (41.8%), diabetes mellitus (38.4%), osteoporosis (17.6%), and atrial fibrillation (2.3%), which all occurred at significantly higher rates in EOD individuals than in those who did not develop EOD (*p* < 0.001) (Table 1).

In the Older group, those who developed LOD were older (LOD 74.2 ± 6.6 years vs. non-LOD 68.3 ± 5.4 years, *p* < 0.001) and more likely to be female (LOD 67.4% vs. non-LOD 53.5%, *p* < 0.001) than those who did not develop LOD. The most prevalent risk factor in LOD patients was hypertension (72.7%), followed by diabetes mellitus (52.1%), hyperlipidemia (50.9%), osteoporosis (47.7%), and atrial fibrillation (4.8%), which all occurred at significantly higher rates in LOD individuals than in those who did not develop LOD (*p* < 0.001) (Table 1).

### Risk and protective factors of EOD and LOD

**Fig. 2:**
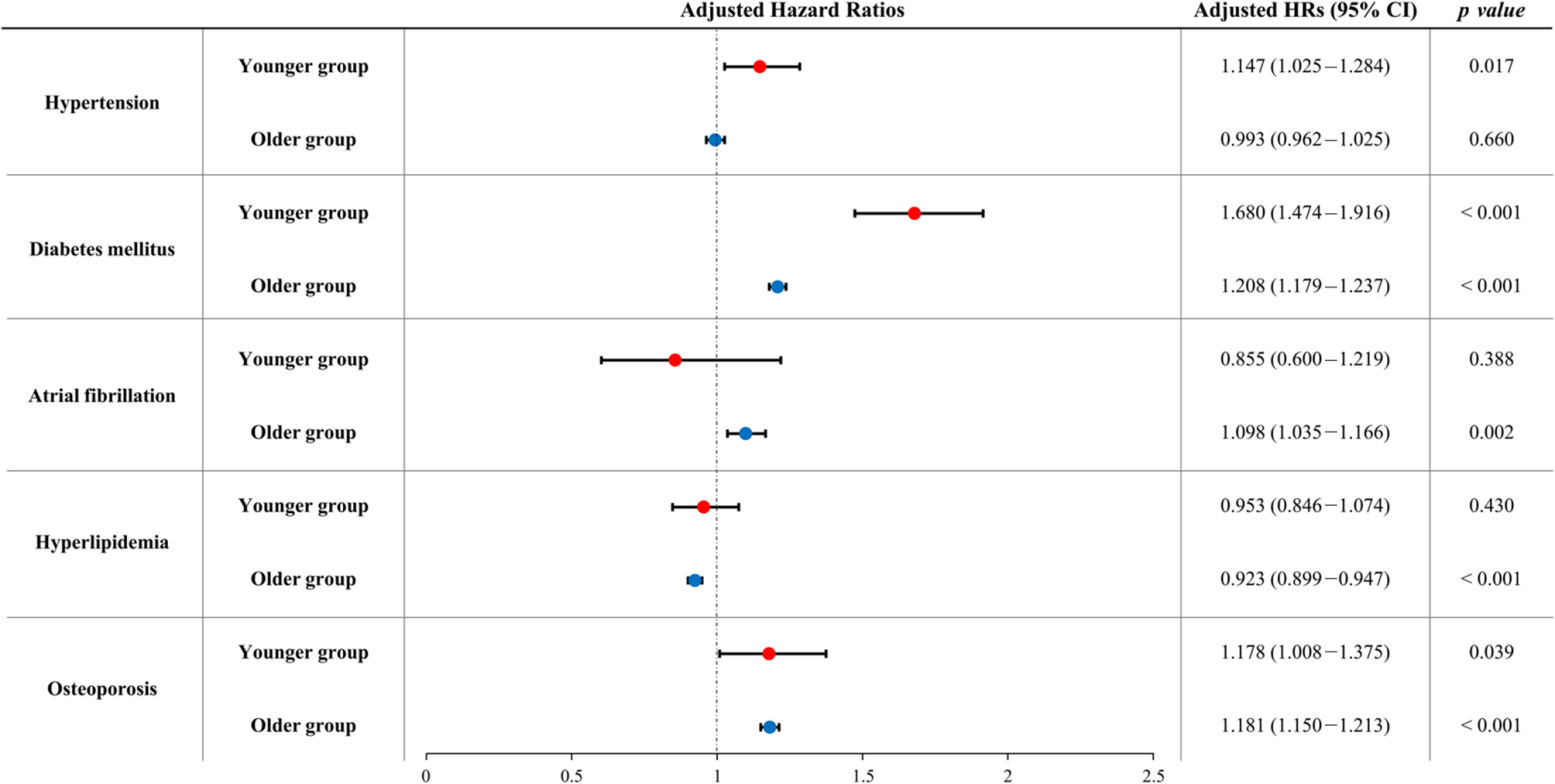
Hazard ratios of each risk factor for development of early-onset dementia and late-onset dementia among the Younger and Older groups, respectively. The hazard ratios are expressed as boxes and the 95% confidence intervals are expressed as limit lines. The multivariate Cox proportional hazard models included age, sex, risk factor of interest (hypertension, diabetes, atrial fibrillation, hyperlipidemia, and osteoporosis), other risk factors in Charlson comorbidity index, socioeconomic status, and residential area as independent variables. Abbreviations: HR, hazard ratios; CI, confidence intervals

The risk factors and protective factors of LOD and EOD were different (Fig. 2 and Supplementary Table 2). In the Younger group, hypertension (HR 1.147, 95% CI 1.025–1.284), diabetes mellitus (HR 1.680, 95% CI 1.474–1.916), and osteoporosis (HR 1.178, 95% CI 1.008–1.375) increased risk of EOD development.

In the Older group, diabetes mellitus (HR 1.208, 95% CI 1.179–1.237), atrial fibrillation (HR 1.098, 95% CI 1.035–1.166), and osteoporosis (HR 1.181, 95% CI 1.150–1.213) increased risk of LOD development. On the other hand, hyperlipidemia was protective against developing LOD (HR 0.923, 95% CI 0.899–0.947).

### Risk and protective factors of mortality among EOD and LOD patients

All-cause mortality and related factors among EOD and LOD are shown in Table 2. The Kaplan–Meier survival curves, starting from dementia diagnosis, are presented in Supplementary Fig. 1 for patients with EOD and LOD. There was a higher death rate in the LOD group compared to the EOD group. During the 11-year follow-up period, 442 (18.7%) died among 2,368 EOD patients, while 12,969 (39.4%) died among 32,893 LOD patients. However, after controlling for age, sex, risk factors of interest, other risk factors of Charlson comorbidity index, socioeconomic status, and residential area, EOD showed a higher influence on mortality than did LOD (HR 2.545, 95% CI: 2.278–2.841, ref. LOD) (Supplementary Fig. 1).

**Fig. 3:**
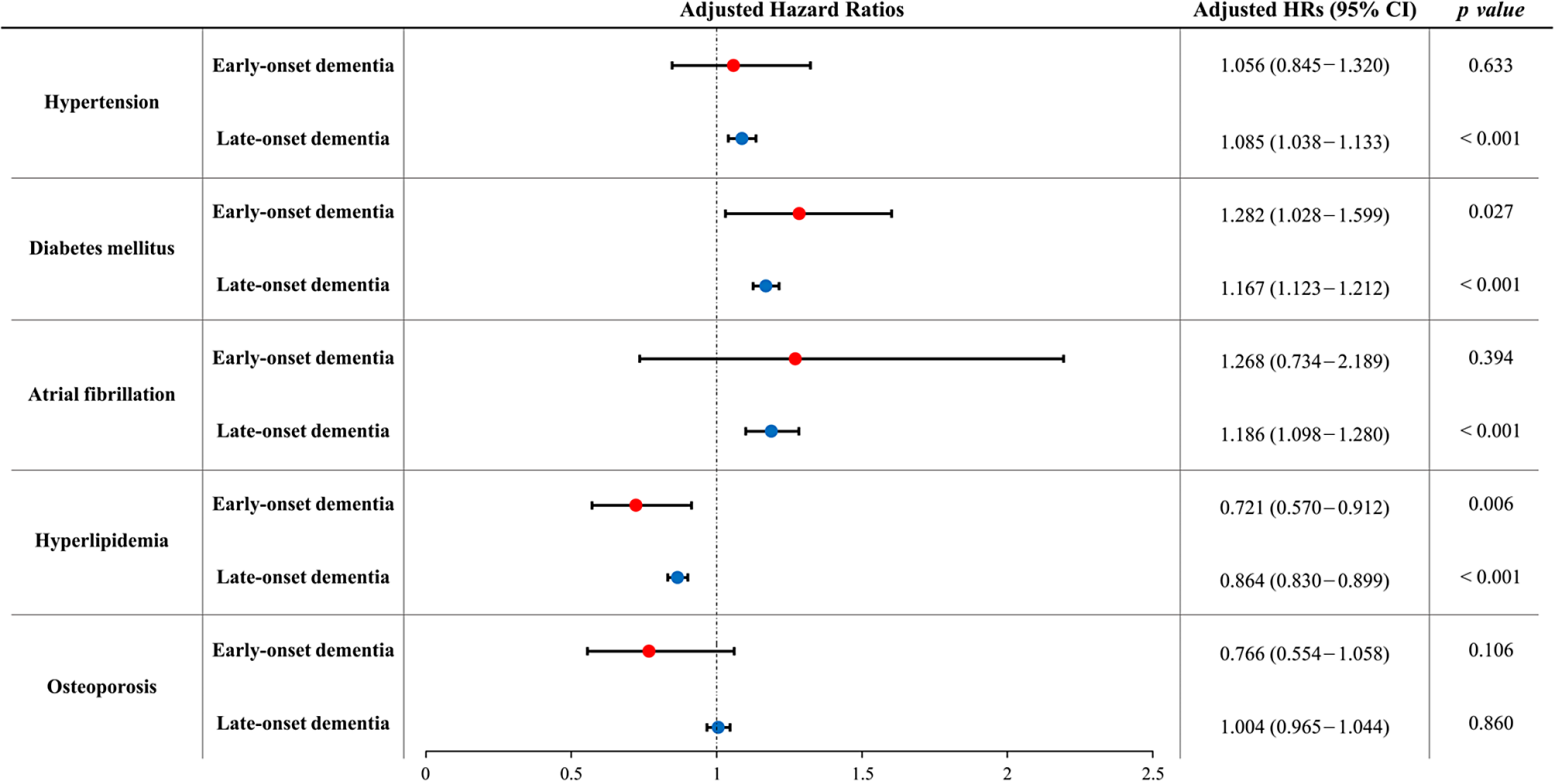
Hazard ratios of each risk factor for all-cause mortality among early-onset dementia and late-onset dementia groups. The hazard ratios are expressed as boxes and the 95% confidence intervals are expressed as limit lines. Abbreviations: HR, hazard ratios; CI, confidence intervals. The multivariate Cox proportional hazard models included age, sex, risk factor of interest (hypertension, diabetes, atrial fibrillation, hyperlipidemia, and osteoporosis), other risk factors in Charlson comorbidity index, socioeconomic status, and residential area as independent variables

The risk factors affecting the mortality among EOD and LOD were different (Fig. 3 and Supplementary Table 3). In EOD patients, diabetes mellitus increased the risk of death (HR 1.282, 95% CI 1.028–1.599), while hyperlipidemia protected against death (HR 0.721, 95% CI 0.570 – 0.912).

In contrast, in LOD patients, hypertension (HR 1.085, 95% CI 1.038–1.133), diabetes mellitus (HR 1.167, 95% CI 1.123–1.212), and atrial fibrillation (HR 1.186, 95% CI 1.098–1.280) increased risk of death. Having hyperlipidemia protected against death in LOD patients (HR 0.864, 95% CI 0.830–0.899).

## Discussion

In this 11-year longitudinal observation study using Korean National cohort data, we investigated how common medical risk factors were associated with the development and mortality of EOD and LOD, respectively. Our study showed that the factors contributing to dementia development and mortality vary according to onset age. Diabetes mellitus and osteoporosis increased the risk of developing both EOD and LOD. Hypertension increased risk of EOD, while atrial fibrillation increased risk of LOD. Hyperlipidemia was a protective factor against development of LOD. The factors leading to death among dementia patients also differed according to the onset age. Diabetes mellitus increased mortality in both EOD and LOD patients, while hypertension and atrial fibrillation were associated with increased mortality among LOD patients. Hyperlipidemia was related to reduced mortality among EOD and LOD patients. Taken together, our findings demonstrate that the onset age matters when considering the effects of risk factors on dementia development and mortality. Therefore, the age of onset is important when establishing strategies for dementia prevention and increasing survival among dementia patients.

Sex distribution varied according to the onset age. The proportion of males was higher among EOD patients (52.2%), whereas the proportion of females was higher among LOD patients (67.4%) compared to the non-dementia counterpart in each age category. It is well known that females are at risk for LOD. However, it has been reported that there is no female predominance in EOD [26]. This could potentially be related to a protective effect of estrogen in younger women [27], or inversely, the higher prevalence of traumatic brain injuries and alcohol-related dementia in males under the age of 65 [28].

Additionally, although LOD showed a higher number of deaths, after adjusting for age, sex, and potential risk factors, having dementia at a young age (EOD) contributed to a higher risk of death compared to having dementia at an older age (LOD) (HR 1.607). This implies that mortality in EOD is driven by dementia itself, whereas mortality in LOD might have been driven by other age-related factors. A previous study indicated that early-onset Alzheimer’s disease patients had higher all-cause mortality when compared to the general population [29]. However, there is a scarcity of population-based studies examining whether the all-cause mortality in dementia patients differs according to age. Although the mechanisms have not been fully elucidated, the pathological burden of neurodegeneration in the brain may vary according to onset age.

Our study showed that diabetes mellitus was a significant contributor to dementia development and mortality in dementia patients in both the Younger and Older groups, which is consistent with previous studies [30–33]. The mechanism by which diabetes mellitus influences dementia development and the mortality of dementia patients may be multifactorial. Previous studies have indicated that the systemic influence of hyperglycemia induces insulin resistance, which impairs insulin signaling in the brain and causes cognitive decline [34–36]. Also, inflammatory mediators and dysregulation of the hypothalamic-pituitary-adrenal axis might also have a role in the development of cognitive impairment in patients with diabetes [32, 37–39]. As diabetes mellitus increases the mortality among dementia patients [40], both EOD and LOD patients with diabetes mellitus are recommended to be regularly screened for cognitive decline.

Osteoporosis was a risk factor for dementia in both the Younger group and the Older group. Osteoporosis frequently accompanies dementia, as both conditions are closely associated with aging [41]. A Swedish national longitudinal study has shown that dementia patients had a higher prevalence of osteoporotic fractures [42]. A population-based cohort analysis in Taiwan and a Danish study have also suggested that osteoporosis is a risk factor for dementia [9, 43]. Although the underlying mechanisms for the high comorbidity rate between dementia and osteoporosis have not been elucidated, the changes in estrogen, immunological factors, or bone-derived proteins may contribute to both bone loss and dementia [44]. Therefore, prevention and treatment of osteoporosis might help prevent the development of both EOD and LOD.

Atrial fibrillation increased the risk of dementia in the Older group and mortality among LOD patients, which is supported by previous studies [45, 46]. Conversely, no significant association was found in the Younger group. Previous studies based on the Korean NHIS-Senior cohort also demonstrated that atrial fibrillation was a risk factor for dementia in the elderly population aged over 60 years [6, 47]. However, Kim et al. have shown that atrial fibrillation had a greater impact on the development of dementia in the younger individuals (< 65 years old) than in the older individuals (≥ 65 years old) [48]. The discrepancy might be due to differences in the study population. In the previous study [48], participants with valvular heart disease or stroke were excluded, whereas our study included these participants.

Hypertension increased the risk of dementia in the Younger group and increased mortality among LOD patients. Our result is consistent with previous reports showing that midlife hypertension elevated the likelihood of developing dementia [49–54]. Moreover, the risk of dementia associated with high blood pressure decreased with increasing age [55, 56]. Thus, effective management of hypertension can potentially reduce the development of EOD and the mortality associated with LOD [57, 58].

In contrast, hyperlipidemia was a protective factor for dementia in the Older group and reduced mortality in both LOD and EOD patients. The effect of hyperlipidemia on dementia is still controversial. Some studies suggest that high triglyceride levels may be associated with an increased risk of dementia [59, 60], whereas others have not found a significant association between hyperlipidemia and cognitive decline [58, 61]. These inconsistencies may be explained by the fact that the relationship between dyslipidemia and dementia is intricate and multifactorial. This finding might have been complicated by lipid-lowering agents, statins, which have protective effects against inflammation and oxidative stress [59] and may reduce the risk of dementia [60, 61]. More studies are needed to determine the associations between hyperlipidemia and dementia.

### Limitations

This study had several limitations. First, although ICD-10 codes were used to define diseases in our study, potential errors may occur due to imprecise coding. Second, we could not evaluate all factors potentially associated with dementia due to a lack of data. Further studies are needed on the effects of risk factors such as education, smoking, alcohol abuse, depression, physical inactivity, hearing loss, head injury, and air pollution, which were lacking in the NHIS database. Third, our study did not consider the treatment of individual risk factors that might modify the association between risk factors and dementia or mortality. Furthermore, because our research objective was to find mortality risk factors in EOD and LOD, we did not analyze whether the impact of risk factors on mortality is increased in the presence of dementia. Future research should provide additional insight into which risk factors should be more tightly controlled, especially in dementia patients of each age group. Finally, for this study, we extracted 20% of the Korean population from the NHIS database due to limitations in handling data volume for analyses. While this may restrict the generalizability of these findings to the entire Korean population, we minimized the extraction bias by using a systematic stratified random sampling method. Nevertheless, the strength of our study is that it is the first population-based study investigating the factors of dementia development and mortality according to onset age.

## Conclusions

In conclusion, the factors influencing the development of dementia and mortality differ between EOD and LOD. It is crucial to establish age-specific strategies to address these factors. Our findings offer a valuable contribution towards devising prevention strategies for dementia development and survival according to the age of onset. Managing age-related risk factors through public health interventions can mitigate both the development and mortality associated with dementia.

### Electronic supplementary material

Below is the link to the electronic supplementary material.

Supplementary Material 1

## Data Availability

No datasets were generated or analysed during the current study.
