# Supplementary material for: Effects of risk factors on the development and mortality of early- and late-onset dementia: an 11-year longitudinal nationwide population-based cohort study in South Korea
Source: Alzheimers Res Ther. 2024 Apr 25;16:92. doi: 10.1186/s13195-024-01436-5 (PMC11044300; doi:10.1186/s13195-024-01436-5)
Supplement: Supplementary file 1 — Supplementary Material 1 [file 13195_2024_1436_MOESM1_ESM.docx]

**Supplementary Table 1.** **ICD-10 and ATC codes used to identify dementia individuals**

| **ICD codes to define dementia patients** |
| --- |
| Dementia in Alzheimer disease (F00.0, F00.1, F00.2, F00.9)  Alzheimer disease (G30.0, G30.1, G30.8, and G30.9) |
| **ATC codes of dementia medication** |
| 148602ATB, 148601ATB, 148602ATD, 148601ATD, 148603ATB, 643402ATD, 643401ATD, 385203ATR, 385204ATR, 385205ATR, 385204ACR, 385203ACR, 385205ACR, 224507CPC, 224506CPC, 224503ACH, 224501ACH, 224508CPC, 224504ACH, 224505ACH, 190004ATB, 190001ATB, 190002ASY, 190004ATD, 190003ATD, 190001ALQ, 190030ASY, 190031ALQ |

* Abbreviations: ICD-10, International Statistical Classification of Diseases and Related Health Problems, 10th revision; ATC, Anatomical Therapeutic Chemical

**Supplementary Table 2. Hazard ratios of each risk factor for development of early-onset dementia and late-onset dementia among the Younger and Older groups, respectively***

| **Age group** | **Risk factors** | **Total (n)** | **Dementia (n)** | **%** | **Adjusted Hazard Ratios^†^** | ***p* value** |
| --- | --- | --- | --- | --- | --- | --- |
| **Younger group** | **Hypertension +** | 115,954 | 1,057 | 0.9 | 1.147 (1.025 – 1.284) | 0.017 |
|  | **Hypertension ‒** | 115,954 | 620 | 0.5 | 1.00 (reference) |  |
| **Older group** | **Hypertension +** | 82,899 | 10,134 | 12.2 | 0.993 (0.962 – 1.025) | 0.660 |
|  | **Hypertension ‒** | 82,899 | 8,992 | 10.8 | 1.00 (reference) |  |
| **Younger group** | **Diabetes mellitus +** | 89,105 | 910 | 1.0 | 1.680 (1.474 – 1.916) | < 0.001 |
|  | **Diabetes mellitus ‒** | 89,105 | 411 | 0.5 | 1.00 (reference) |  |
| **Older group** | **Diabetes mellitus +** | 117,141 | 17,142 | 14.6 | 1.208 (1.179 – 1.237) | < 0.001 |
|  | **Diabetes mellitus ‒** | 117,141 | 14,708 | 12.6 | 1.00 (reference) |  |
| **Younger group** | **Atrial fibrillation +** | 5,269 | 54 | 1.0 | 0.855 (0.600 – 1.219) | 0.388 |
|  | **Atrial fibrillation ‒** | 26,345 | 177 | 0.7 | 1.00 (reference) |  |
| **Older group** | **Atrial fibrillation +** | 9,940 | 1,564 | 15.7 | 1.098 (1.035 – 1.166) | 0.002 |
|  | **Atrial fibrillation ‒** | 49,700 | 7,655 | 15.4 | 1.00 (reference) |  |
| **Younger group** | **Hyperlipidemia +** | 123,711 | 989 | 0.8 | 0.953 (0.846 – 1.074) | 0.430 |
|  | **Hyperlipidemia ‒** | 123,711 | 656 | 0.5 | 1.00 (reference) |  |
| **Older group** | **Hyperlipidemia +** | 98,103 | 15,039 | 15.3 | 0.923 (0.899 – 0.947) | < 0.001 |
|  | **Hyperlipidemia ‒** | 98,103 | 16,021 | 16.3 | 1.00 (reference) |  |
| **Younger group** | **Osteoporosis +** | 49,302 | 417 | 0.8 | 1.178 (1.008 – 1.375) | 0.039 |
|  | **Osteoporosis ‒** | 49,302 | 293 | 0.6 | 1.00 (reference) |  |
| **Older group** | **Osteoporosis +** | 65,050 | 14,801 | 22.8 | 1.181 (1.150 – 1.213) | < 0.001 |
|  | **Osteoporosis ‒** | 65,050 | 9,802 | 15.1 | 1.00 (reference) |  |

^*^ Cox Proportional Hazard Regression Analysis

^†^Adjusted for age, sex, risk factors of interest (hypertension, diabetes, atrial fibrillation, hyperlipidemia, and osteoporosis), other risk factors in Charlson comorbidity index, socioeconomic status, and residential area

**Supplementary Table 3. Hazard ratios of each risk factor for all-cause mortality among early-onset dementia and late-onset dementia, respectively^*^**

| **Age group** | **Risk factors** | **Dementia patients (n)** | **Death (n)** | **(%)** | **Total (Person-years)** | **Adjusted Hazard Ratios^†^** | ***p* value** |
| --- | --- | --- | --- | --- | --- | --- | --- |
| **EOD** | **Hypertension +** | 1,057 | 224 | 21.2 | 10,828 | 1.056 (0.845 – 1.32) | 0.633 |
|  | **Hypertension ‒** | 1,311 | 218 | 16.6 | 13,775 | 1.00 (reference) |  |
| **LOD** | **Hypertension +** | 23,901 | 9,782 | 40.9 | 218,889 | 1.085 (1.038 – 1.133) | < 0.001 |
|  | **Hypertension ‒** | 8,992 | 3,187 | 35.4 | 84,450 | 1.00 (reference) |  |
| **EOD** | **Diabetes mellitus +** | 910 | 205 | 22.5 | 9,240 | 1.282 (1.028 – 1.599) | 0.027 |
|  | **Diabetes mellitus ‒** | 1,458 | 237 | 16.3 | 15,363 | 1.00 (reference) |  |
| **LOD** | **Diabetes mellitus +** | 17,143 | 6,832 | 39.9 | 156,378 | 1.167 (1.123 – 1.212) | < 0.001 |
|  | **Diabetes mellitus ‒** | 15,750 | 6,137 | 39.0 | 146,961 | 1.00 (reference) |  |
| **EOD** | **Atrial fibrillation +** | 54 | 16 | 29.6 | 532 | 1.268 (0.734 – 2.189) | 0.394 |
|  | **Atrial fibrillation ‒** | 2,314 | 426 | 18.4 | 24,071 | 1.00 (reference) |  |
| **LOD** | **Atrial fibrillation +** | 1,564 | 752 | 48.1 | 13,506 | 1.186 (1.098 – 1.280) | < 0.001 |
|  | **Atrial fibrillation ‒** | 31,329 | 12,217 | 39.0 | 289,833 | 1.00 (reference) |  |
| **EOD** | **Hyperlipidemia +** | 989 | 179 | 18.1 | 10,249 | 0.721 (0.570 – 0.912) | 0.006 |
|  | **Hyperlipidemia ‒** | 1,379 | 263 | 19.1 | 14,354 | 1.00 (reference) |  |
| **LOD** | **Hyperlipidemia +** | 16,756 | 5,991 | 35.8 | 154,441 | 0.864 (0.830 – 0.899) | < 0.001 |
|  | **Hyperlipidemia ‒** | 16,137 | 6,978 | 43.2 | 148,899 | 1.00 (reference) |  |
| **EOD** | **Osteoporosis +** | 417 | 54 | 12.9 | 4,375 | 0.766 (0.554 – 1.058) | 0.106 |
|  | **Osteoporosis ‒** | 1,951 | 388 | 19.9 | 20,228 | 1.00 (reference) |  |
| **LOD** | **Osteoporosis +** | 15,689 | 5,806 | 37.0 | 146,469 | 1.004 (0.965 – 1.044) | 0.860 |
|  | **Osteoporosis ‒** | 17,204 | 7,163 | 41.6 | 156,871 | 1.00 (reference) |  |

Abbreviations: LOD, Late-onset dementia; EOD, Early-onset dementia

*Cox Proportional Hazard Regression Analysis

^†^Adjusted for age, sex, risk factors of interest (hypertension, diabetes, atrial fibrillation, hyperlipidemia, and osteoporosis), other risk factors in Charlson comorbidity index, socioeconomic status, and residential area


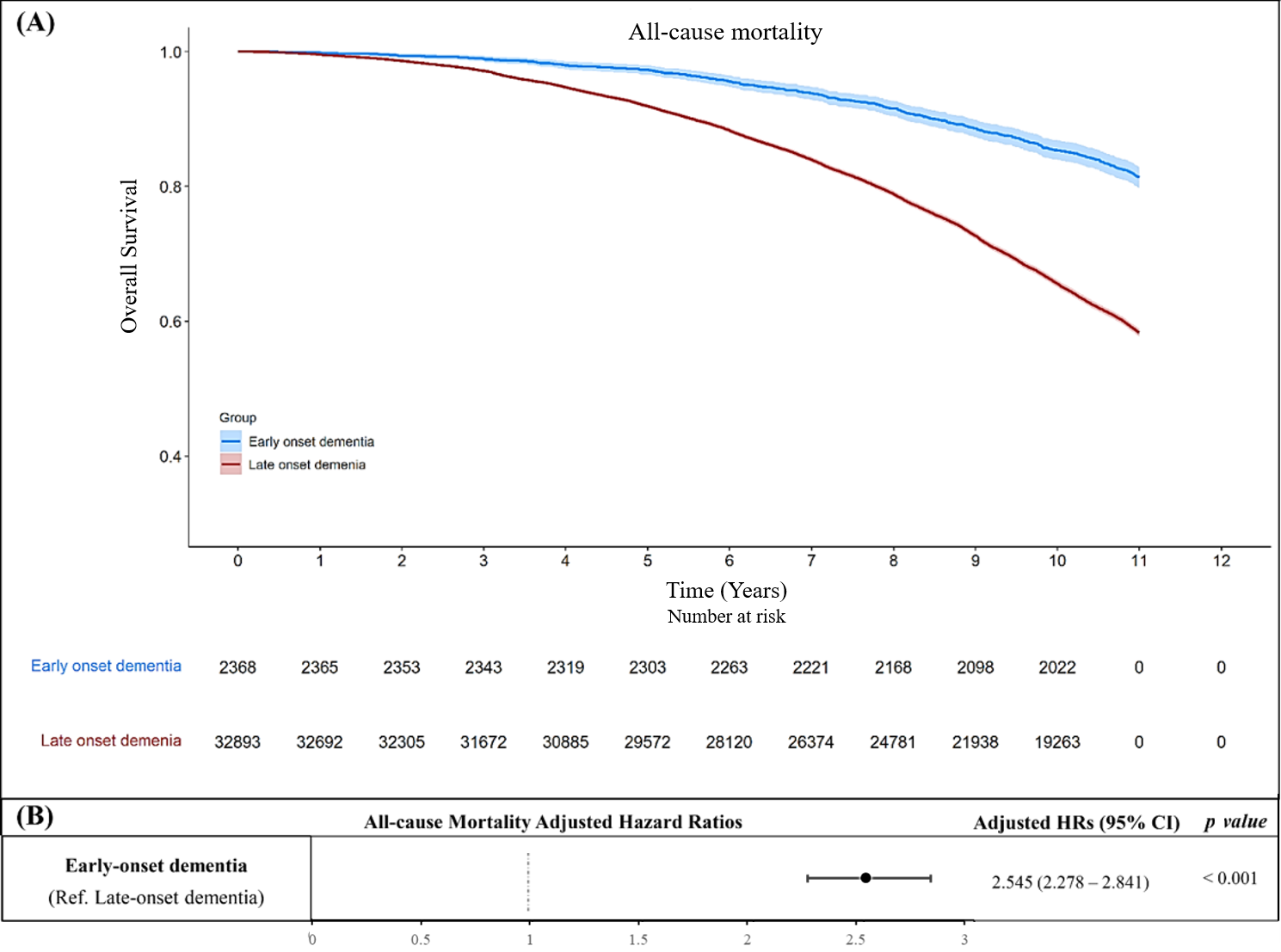


**Supplementary Figure 1**. **(A) Kaplan–Meier survival curves from time of dementia diagnosis in early-onset dementia and late-onset dementia individuals. (B) Hazard ratio of early-onset dementia (compared to late-onset dementia) for mortality.** (A) Kaplan–Meier survival curves from time of dementia diagnosis were generated in early-onset dementia and late-onset dementia groups, respectively. (B) The adjusted hazard ratio for mortality in early-onset dementia group compared to late-onset dementia group was calculated using a multivariate Cox proportional hazard model including dementia group (early-onset vs late onset), age, sex, risk factors (hypertension, diabetes, atrial fibrillation, hyperlipidemia, and osteoporosis), other risk factors in Charlson comorbidity index, socioeconomic status, and residential area as independent variables.
